# Circulating Tumor DNA in Muscle-Invasive Bladder Cancer: Implications for Prognosis and Treatment Personalization

**DOI:** 10.3390/cancers17121908

**Published:** 2025-06-08

**Authors:** Stamatios Katsimperis, Lazaros Tzelves, Georgios Feretzakis, Themistoklis Bellos, Ioannis Tsikopoulos, Nikolaos Kostakopoulos, Andreas Skolarikos

**Affiliations:** 1 Second Department of Urology, National and Kapodistrian University of Athens, Sismanogleio Hospital, 15126 Athens, Greece; 2 School of Science and Technology, Hellenic Open University, 26335 Patras, Greece; 3 Royal National Orthopaedic Hospital, London W1W 5AQ, UK; 4 First Department of Urology, Metropolitan General Hospital, 15562 Athens, Greece

**Keywords:** muscle-invasive bladder cancer, circulating tumor DNA, liquid biopsy, cell-free DNA, biomarkers, risk stratification, adjuvant immunotherapy, precision oncology, tumor-informed assays, urothelial carcinoma, molecular diagnostics, treatment personalization, bladder cancer surveillance

## Abstract

Muscle-invasive bladder cancer (MIBC) is a biologically aggressive disease with high recurrence rates, despite advances in surgical and systemic therapies. Circulating tumor DNA (ctDNA), a tumor-specific fraction of cell-free DNA, has emerged as a promising non-invasive biomarker for the real-time assessment of tumor burden, treatment response, and minimal residual disease (MRD). This review explores the biological basis, detection technologies, and clinical utility of ctDNA in MIBC, highlighting its role in preoperative risk stratification, postoperative surveillance, and personalized decision-making for adjuvant and systemic therapies. We critically examine current evidence from pivotal trials and ongoing studies that support ctDNA’s prognostic and predictive value. Additionally, we discuss emerging applications, including ctDNA-guided immunotherapy, integration with imaging and molecular data, and potential to inform bladder-sparing strategies. While ctDNA presents technical and logistical challenges, its incorporation into prospective clinical workflows promises to enhance precision oncology and improve outcomes in patients with MIBC.

## Simple Summary

Muscle-invasive bladder cancer is a clinically aggressive disease with a high risk of recurrence despite optimal treatment. Current methods for assessing treatment response and detecting residual disease rely primarily on imaging and pathology, which may not accurately reflect the presence of microscopic cancer. Circulating tumor DNA, detectable through a blood test, represents a promising non-invasive biomarker that can provide real-time information about tumor burden and treatment effectiveness. This review discusses the biological basis, detection methods, and clinical applications of circulating tumor DNA in the management of muscle-invasive bladder cancer. It highlights its potential to improve risk stratification, guide postoperative treatment decisions, and enable personalized surveillance strategies. Incorporating this approach into clinical practice may support more precise and timely interventions, ultimately enhancing patient outcomes and informing future research in precision oncology.

## 1. Introduction

Muscle-invasive bladder cancer (MIBC) represents a biologically aggressive subset of urothelial carcinoma, accounting for approximately 25% of newly diagnosed bladder cancer cases [1]. Despite advances in surgical techniques and systemic therapies, MIBC continues to be associated with significant morbidity and mortality, with a five-year overall survival rate ranging from 50% to 70% depending on stage and histologic features [2,3]. The current standard of care for non-metastatic MIBC includes radical cystectomy (RC) combined with cisplatin-based neoadjuvant chemotherapy (NAC) for eligible patients, offering modest improvements in survival. However, the response to NAC is heterogeneous, with only a fraction achieving complete tumor eradication, and many continue to face disease relapse even after undergoing definitive treatment [4].

A critical challenge in managing MIBC is the lack of reliable, non-invasive biomarkers that can provide real-time insights into tumor dynamics, guide perioperative treatment decisions, and predict individual patient outcomes. Traditional imaging and tissue-based histopathology often fail to detect minimal residual disease (MRD) or accurately stratify patients based on recurrence risk. This underscores the urgent need for novel biomarkers that can enhance precision oncology approaches in MIBC.

Circulating tumor DNA (ctDNA), a tumor-derived fraction of cell-free DNA (cfDNA) shed into the bloodstream, has emerged as a promising tool in this context. ctDNA reflects the genetic landscape of the tumor, carrying somatic mutations, copy number alterations, and other tumor-specific aberrations. With a short half-life and dynamic nature, ctDNA allows for the real-time monitoring of tumor burden, assessment of treatment response, and early detection of relapse [5,6,7,8,9,10]. Unlike tissue biopsies, ctDNA analysis offers a non-invasive method of capturing tumor heterogeneity and clonal evolution, particularly valuable in MIBC, which is characterized by a high degree of molecular complexity.

Recent studies have demonstrated the potential of ctDNA to refine risk stratification before and after radical cystectomy, monitor MRD, and guide the use of adjuvant therapies such as immune checkpoint inhibitors [11,12]. The predictive value of ctDNA has also been highlighted in landmark trials, such as IMvigor010, where ctDNA-positive patients exhibited significantly improved outcomes when treated with adjuvant immunotherapy [13]. Despite these promising results, the integration of ctDNA into routine clinical practice remains limited by technical, biological, and logistical challenges.

This review aims to explore the clinical utility of ctDNA in MIBC, focusing on its role in preoperative risk assessment, post-cystectomy surveillance, and the personalization of perioperative treatment strategies. By synthesizing current evidence, we highlight how ctDNA may transform the management of MIBC through enhanced prognostication and individualized therapy.

## 2. Biological Basis and Detection of Circulating Tumor DNA

Circulating tumor DNA (ctDNA) is a component of cell-free DNA (cfDNA) that originates specifically from tumor cells. In cancer patients, cfDNA is released into the bloodstream primarily through apoptosis and necrosis of tumor cells, as well as through potential active secretion mechanisms [14,15,16]. These DNA fragments, typically short and highly fragmented, contain tumor-specific genetic and epigenetic alterations such as point mutations, copy number variations, rearrangements, and methylation patterns. The detection of ctDNA offers a non-invasive window into the molecular landscape of the tumor, providing critical insights into tumor burden, treatment response, and disease progression.

In MIBC, ctDNA levels are closely associated with the tumor’s biological activity treatment and burden. One of the key advantages of ctDNA over traditional tissue biopsy is its ability to capture the dynamic nature of the disease in real time. This is due to the short half-life of ctDNA, estimated between 15 min and 2 hours, which allows for the immediate reflection of changes in tumor activity, whether due to natural disease progression or therapeutic intervention [17,18]. Unlike static, spatially limited tissue biopsies, ctDNA offers a broader and more temporally sensitive perspective, as it represents DNA fragments shed from multiple tumor sites, including both primary and metastatic lesions. This aspect is especially relevant in MIBC, a disease characterized by considerable molecular heterogeneity and clonal evolution, particularly under therapeutic pressure.

The biological variability of ctDNA, shaped by factors such as tumor burden, vascular supply, and cellular turnover, poses significant challenges for reliable detection. Compounding this issue is the fact that ctDNA often represents only a small percentage (sometimes < 0.01%) of the total cell-free DNA in the peripheral blood, and its identification typically relies on prior knowledge of tumor-specific mutations, which is not always readily available [17,19]. The accurate detection of ctDNA at such low concentrations is critical for clinical applications, including the assessment of MRD and early recurrence.

Two distinct methodologies are commonly used for analyzing ctDNA: tumor-informed and tumor-agnostic techniques. Tumor-informed testing involves an initial, detailed genetic analysis of a patient’s tumor tissue to pinpoint unique somatic mutations. These identified mutations are subsequently monitored in blood samples using personalized assays, which offer exceptional sensitivity and accuracy, making them highly effective for identifying MRD. Such tailored assays, like Signatera™, have demonstrated considerable clinical relevance in monitoring MIBC, particularly around the time of radical cystectomy. The main limitation of this method lies in its reliance on tumor tissue availability and the time-intensive process of developing customized tests [20]. Another drawback is that, although targeting patient-specific mutations enhances specificity, detection is confined to variants present in the initially analyzed tumor tissue, potentially overlooking new or evolving mutations [21,22].

On the other hand, tumor-agnostic assays operate using standardized gene panels that focus on frequently mutated regions commonly found in bladder cancer. These tests do not require prior tumor sequencing, enabling quicker deployment and faster results. However, they typically exhibit reduced sensitivity and may produce false-positive results, especially when ctDNA is present at very low levels. While tumor-agnostic methods can be advantageous when rapid testing is needed or tumor tissue is unobtainable, they generally lack the precision of tumor-informed strategies, particularly when used for detecting subtle traces of residual disease [23]. To summarize the key differences between these two methodologies, Table 1 provides a comparative overview of their respective advantages and limitations.

The technological platforms employed for ctDNA analysis include digital droplet PCR (ddPCR) and next-generation sequencing (NGS). Digital droplet PCR is a highly sensitive technique capable of detecting specific, low-frequency mutations with precision. Its affordability and rapid processing make it well suited for a focused analysis of mutations already identified in a patient’s tumor. However, because ddPCR targets only a limited set of known alterations, it is not appropriate for comprehensive genomic assessment or the discovery of novel mutations [24,25].

NGS, on the other hand, allows for a comprehensive analysis of a wide range of genetic alterations, including single nucleotide variants, insertions, deletions, and structural rearrangements. With the integration of unique molecular identifiers and advanced error correction algorithms, the sensitivity of NGS-based ctDNA assays has significantly improved, making them capable of detecting mutations with variant allele frequencies approaching those achievable by ddPCR. Although more expensive and requiring more extensive bioinformatics support, NGS offers the advantage of broader mutation detection, which is particularly beneficial in a heterogeneous disease like MIBC [26].

Emerging methodologies such as methylation-based ctDNA detection are also gaining attention. Tumor-derived ctDNA carries specific methylation patterns that differ from non-tumor DNA. These methylation signatures can potentially enhance detection sensitivity, particularly in tumors with low mutational burdens, and may allow for the identification of ctDNA in settings where mutation-based detection is challenging. Preliminary studies in bladder cancer suggest that methylation analysis may complement traditional mutation-based ctDNA assays, expanding the utility of liquid biopsies in this context [26].

Despite the promise of ctDNA in transforming the management of MIBC, several challenges remain. The variability in ctDNA shedding between patients, influenced by tumor biology and individual patient characteristics, complicates the interpretation of ctDNA results. Furthermore, the lack of standardized protocols across laboratories limits the comparability of findings and hinders the broader implementation of ctDNA testing in clinical practice. False positives, particularly due to clonal hematopoiesis, require careful differentiation to avoid misinterpretation of ctDNA results. Additionally, the high cost and technical demands of personalized ctDNA assays present practical barriers to widespread adoption, especially in resource-limited settings [27,28].

In addition to plasma-based analysis, urine-derived ctDNA has also gained attention as a promising non-invasive biomarker, particularly for bladder cancer. Given the direct interaction between urine and the urothelial tract, urine samples may yield higher concentrations of tumor DNA, especially in localized disease. Studies have shown that urine ctDNA can achieve comparable or even superior sensitivity and specificity to plasma ctDNA for detecting bladder cancer mutations, notably telomerase reverse transcriptase (TERT) promoter mutations, which have demonstrated high specificity [29,30]. Furthermore, urine sampling offers practical advantages, such as ease of collection and the potential for more frequent, cost-effective monitoring, making it a valuable complement to plasma ctDNA in both detection and surveillance strategies.

## 3. ctDNA in Preoperative Risk Assessment

As discussed in the previous section, ctDNA’s ability to provide a dynamic and highly specific insight into tumor burden and biology has positioned it as a candidate biomarker for clinical use in MIBC. The preoperative period, especially the time before radical cystectomy, represents a critical opportunity to utilize ctDNA for personalized risk assessment. This phase of treatment often involves decisions regarding the use of NAC and surgical planning, both of which could be optimized with more precise biomarkers than those currently available.

Current clinical practice relies heavily on imaging studies, histopathological grading, and clinical staging to determine a patient’s risk profile and guide treatment. However, these traditional tools lack the sensitivity to detect microscopic disease spread or to reflect the biological aggressiveness of the tumor in real time. ctDNA, on the other hand, offers a molecular measure of disease burden that can be assessed non-invasively before surgery, providing clinicians with actionable data to stratify patients more effectively.

Emerging evidence suggests that detectable ctDNA before cystectomy is associated with adverse pathological features and worse clinical outcomes. For example, Christensen et al. demonstrated that patients with preoperative ctDNA positivity exhibited significantly higher rates of recurrence, lower disease-free survival (DFS) and worse OS (*p* = 0.018), with 89% of patients with increased ctDNA eventually having a relapse after cystectomy compared to those without detectable ctDNA [31]. In a subsequent study conducted by the same team at Aarhus University Hospital, ctDNA levels were longitudinally monitored in 68 individuals with MIBC undergoing neoadjuvant treatment. Among these, 24 patients tested ctDNA-positive before starting therapy, and 10 of them relapsed within a year following NAC and cystectomy. By comparison, only one recurrence was noted among the 35 patients who were ctDNA-negative at baseline. Moreover, changes in ctDNA levels over the course of treatment were associated with treatment response; clearance of ctDNA was correlated with tumor downstaging at the time of surgery, suggesting that serial ctDNA monitoring may reflect therapeutic efficacy. The study also highlighted the prognostic value of ctDNA for MRD detection post-surgery: of the 17 patients with detectable ctDNA after cystectomy, the majority (59%) relapsed within a year, and 76% experienced recurrence during the study period. Notably, none of the 47 patients who remained ctDNA-negative during follow-up developed clinical recurrence [32]. These observations were further supported by Lindskrog et al., who conducted an extended follow-up (median 68 months) of the same cohort. Their analysis confirmed that ctDNA positivity before NAC, after NAC, and post-cystectomy remained strongly predictive of both recurrence-free and overall survival. Moreover, patients with undetectable ctDNA at these time points were more likely to achieve pathological downstaging and demonstrated improved survival outcomes [10]. Similarly, Sfakianos et al. demonstrated that ctDNA detection prior to radical cystectomy predicted significantly shorter disease-free survival (median 8 months vs not reached; HR 10.6, 95% CI 1.01–1434; *p* = 0.035). In their study, 40% of patients tested ctDNA-positive before surgery, and none of the ctDNA-negative patients experienced recurrence at the time of analysis. These results highlight the potential of preoperative ctDNA status to identify patients who could benefit from more intensive perioperative treatments, including tailored chemotherapy or participation in neoadjuvant immunotherapy trials [11].

Recent research has emphasized the role of ctDNA as a dynamic biomarker for evaluating neoadjuvant immunotherapy response in MIBC. In the ABACUS trial, a phase II study which enrolled cisplatin-ineligible patients, 63% had detectable ctDNA before receiving atezolizumab, 47% remained positive after treatment, and only 14% had ctDNA post-cystectomy [33]. Patients who were ctDNA-negative at baseline or achieved ctDNA clearance after immunotherapy were more likely to show pathological response and had favorable outcomes, with no relapses observed. Conversely, individuals with detectable ctDNA following surgery faced a markedly higher risk of recurrence (RFS HR = 78, *p* < 0.001) [1]. The NABUCCO trial, investigating neoadjuvant ipilimumab plus nivolumab in high-risk MIBC, similarly found that ctDNA negativity at surgery was associated with 88% progression-free survival at 12 months, compared to 55% in those with detectable ctDNA [34]. Additionally, ctDNA-negative status was significantly correlated with treatment response (*p* < 0.01) and a lower risk of progression (PFS HR = 10.4, 95% CI: 2.9–37.5) [34].

Building on these findings, the ongoing VOLGA trial (NCT04960709), a multicenter phase III study, is examining the combination of neoadjuvant durvalumab, tremelimumab, and gemcitabine/cisplatin chemotherapy in MIBC patients [35].

Importantly, this trial incorporates tumor-informed ctDNA monitoring to investigate whether ctDNA clearance can serve as a surrogate for treatment response and predict pathological complete response at cystectomy. Preliminary results from the safety cohort suggest that patients who were ctDNA-negative or cleared ctDNA had better failure-free survival, while persistent ctDNA positivity was associated with higher rates of pathological upstaging. By integrating ctDNA dynamics into prospective trials like VOLGA, researchers aim to validate its prognostic value, with the goal of adapting perioperative treatment intensity based on early molecular response, potentially guiding both escalation and de-escalation strategies.

## 4. The Role of ctDNA in MRD Monitoring and Personalizing Adjuvant Treatment

Following radical cystectomy, patients with MIBC remain at substantial risk for recurrence, often due to micrometastatic disease that escapes detection at the time of surgery. Identifying MRD postoperatively is essential for guiding adjuvant treatment and improving long-term outcomes. Conventional surveillance methods, based on imaging and clinical evaluation, typically reveal recurrence only once significant tumor burden is present. In contrast, ctDNA enables the molecular detection of MRD, often preceding clinical or radiographic signs of relapse by several months, providing a critical window for earlier intervention.

Postoperative ctDNA positivity has consistently been linked to poor prognosis across multiple studies. Notably, the study by Sfakianos et al., which previously demonstrated the prognostic relevance of preoperative ctDNA, also showed that ctDNA detection after surgery is a strong independent predictor of recurrence. Their findings revealed that patients with detectable ctDNA had significantly worse disease-free survival, with hazard ratios of 6.93 during the MRD window and 23.02 during the surveillance period, underscoring ctDNA’s value for the early identification of high-risk patients [11]. Furthermore, Carrasco et al. reported that ctDNA positivity preceded radiologic recurrence by a median of 6 months, while clearance of ctDNA 4 months post-cystectomy correlated with improved outcomes [36].

Beyond prognostication, ctDNA has emerged as a valuable tool for stratifying patients most likely to benefit from adjuvant therapies. In the IMvigor010 trial, a post hoc analysis revealed that ctDNA-positive patients derived a significant survival benefit from adjuvant immunotherapy with atezolizumab, whereas ctDNA-negative patients did not show the same advantage [13]. While the trial did not show a statistically significant improvement in disease-free survival (DFS) for the overall cohort receiving atezolizumab versus observation, ctDNA status offered critical stratification. Among patients with detectable ctDNA after cystectomy (37% of the biomarker-evaluable population), those who received atezolizumab had markedly improved outcomes compared to observation alone, with a DFS hazard ratio of 0.58 (95% CI: 0.43–0.79) and an overall survival (OS) hazard ratio of 0.59 (95% CI: 0.41–0.86). Conversely, ctDNA-negative patients did not derive the same benefit (DFS HR = 1.14, 95% CI: 0.81–1.62). Furthermore, ctDNA clearance was more frequently observed in the atezolizumab group, with 18.2% clearing ctDNA by 6 weeks post-randomization compared to only 3.8% in the observation arm (*p* = 0.024). Importantly, patients who achieved ctDNA clearance experienced significantly better DFS (HR = 0.26, 95% CI: 0.12–0.56) than those with persistent ctDNA, reinforcing ctDNA’s role not only as a prognostic marker but also as a predictive tool for therapeutic benefit. A subsequent long-term analysis of IMvigor010, with a median follow-up of 46.8 months, confirmed that ctDNA positivity immediately after cystectomy remained a strong predictor of poor OS (median OS: 14.1 months vs. not reached; HR 6.3, 95% CI: 4.3–9.3) [7]. Moreover, a quantitative correlation between ctDNA reduction and survival was observed: patients achieving complete ctDNA clearance had the best outcomes (median OS: 60.0 months), compared to partial reductions of 50–99% (34.3 months) or less than 50% (19.9 months). These findings support the integration of ctDNA analysis into personalized postoperative management strategies.

This stratified approach is now being prospectively tested in the ongoing IMvigor011 trial (NCT04660344), where patients with detectable ctDNA following cystectomy are randomized to receive either atezolizumab or placebo. The main objective is to assess disease-free survival specifically in ctDNA-positive individuals, while those without ctDNA are monitored in a non-interventional arm. This study is among the first to prospectively investigate the use of ctDNA for guiding adjuvant treatment decisions in urothelial cancer [37].

One of the most innovative aspects of ctDNA is its potential to guide surveillance dynamically, moving beyond fixed schedules. Its high negative predictive value allows for safely deferring adjuvant therapy or intensive imaging in ctDNA-negative patients, while enabling early intervention in those with rising ctDNA levels. This approach is being explored in trials like TOMBOLA (NCT04138628), where patients receive atezolizumab only upon ctDNA positivity. Preliminary data suggest a relapse rate of just 3% among ctDNA-negative patients, supporting a “watchful waiting” strategy that minimizes overtreatment [38].

Further, the MODERN trial (NCT05987241) advances this adaptive model. Post-cystectomy patients are stratified by ctDNA status: ctDNA-positive individuals are randomized to receive nivolumab alone or with relatlimab, while ctDNA-negative patients are observed. This study seeks to validate both escalation and de-escalation based on ctDNA, pushing bladder cancer management toward a more individualized, risk-adapted paradigm.

Beyond immunotherapy, ctDNA is also emerging as a valuable tool for guiding targeted therapies. Mutations in the FGFR3 gene, frequently found in a subset of bladder cancer patients, can be detected through ctDNA analysis. These genetic alterations are targetable with FGFR inhibitors, such as erdafitinib, which has shown clinical efficacy in FGFR3-mutant urothelial carcinoma. By identifying FGFR3 mutations non-invasively, ctDNA testing may help select patients for these targeted treatments and monitor their response over time [31,39]. This broadens the clinical relevance of ctDNA, positioning it as a versatile biomarker not only for immunotherapy stratification but also for personalizing molecularly targeted systemic therapy in MIBC.

ctDNA is also being explored as a tool to support bladder-sparing strategies. In patients who achieve both radiologic and molecular complete responses following neoadjuvant therapies, deferring radical cystectomy is under active investigation. Persistent ctDNA clearance in these individuals could indicate durable remission, while the re-emergence of ctDNA may warrant early salvage intervention. Additionally, serial ctDNA monitoring provides valuable insights into tumor evolution and emerging resistance mechanisms, enabling timely adjustments in therapy. This approach has the potential to support mutation-driven treatment strategies, similar to those successfully applied in other solid tumors [40,41].

In summary, ctDNA has become an important tool across multiple stages of MIBC management. Its current applications include the detection of MRD, stratification for adjuvant therapies, and early identification of recurrence. Furthermore, ctDNA facilitates the real-time assessment of treatment response, enables selection of patients for immunotherapy or targeted therapies, and supports emerging bladder-sparing approaches. These evolving uses of ctDNA underscore its growing role in advancing precision oncology for patients with MIBC.

## 5. Challenges and Future Perspectives

While the clinical applications of circulating tumor DNA (ctDNA) in muscle-invasive bladder cancer (MIBC) are expanding rapidly, several challenges must be addressed before ctDNA can be fully integrated into routine clinical practice. Traditionally, decisions regarding adjuvant therapy have relied on pathological features such as extravesical extension, lymphovascular invasion, and nodal involvement [42]. However, these criteria are often inadequate in accurately predicting recurrence risk, leading to potential over- or under-treatment. The integration of ctDNA into clinical decision-making offers a more biologically precise method for identifying patients who are most likely to benefit from systemic therapies, thereby improving treatment outcomes while minimizing unnecessary exposure to toxicities.

Despite its promise, ctDNA testing faces technical, biological, clinical, and logistical hurdles. A primary technical challenge is the lack of standardization across ctDNA assays. The wide variety of detection platforms—ranging from digital droplet PCR to diverse next-generation sequencing (NGS) approaches—results in variability in sensitivity, specificity, and reporting standards [25,26]. Differences in sample processing, sequencing depth, and bioinformatics pipelines can significantly impact ctDNA results, complicating cross-study comparisons and clinical decision-making [43,44,45]. Establishing standardized protocols and quality control measures is essential to ensure reproducibility and reliability across laboratories and clinical settings.

Another critical issue is the biological variability in ctDNA shedding. Not all tumors release ctDNA at detectable levels, and shedding rates can differ based on tumor size, vascularization, location, and metabolic activity. This can lead to false-negative results, particularly in patients with low tumor burden or in early postoperative stages. Additionally, clonal hematopoiesis, a benign age-related condition, can introduce false positives due to non-tumor-derived mutations detected in ctDNA assays [46]. Distinguishing tumor-specific ctDNA from non-tumor cfDNA remains a key focus of ongoing research.

From a clinical standpoint, defining actionable thresholds for ctDNA positivity and clearance is imperative. While correlations between ctDNA dynamics and outcomes have been established, universally accepted cut-offs for clinically significant ctDNA results are lacking. Furthermore, the optimal timing, frequency, and interpretation of serial ctDNA testing need refinement. Although longitudinal monitoring may offer greater insight than single time-point assessments, the logistics and costs of frequent testing must be justified by clear clinical benefits. The significance of partial ctDNA responses also remains uncertain, necessitating further study.

Cost and accessibility present additional barriers to widespread adoption. Tumor-informed ctDNA assays, while offering high specificity, are resource-intensive and may not be readily available in all healthcare systems. Broader implementation will depend on efforts to reduce costs, streamline assay development, and demonstrate cost-effectiveness in improving patient outcomes.

In addition to these challenges, emerging innovations in ctDNA analysis are enhancing its potential clinical value. One such advancement is the Molecular Tumor Burden Index (mTBI), a quantitative metric derived from ctDNA that estimates the total burden of tumor-derived mutations. Preliminary research indicates that mTBI may serve as a robust prognostic indicator, correlating with recurrence risk and therapeutic response, especially following immunotherapy [47,48]. Integrating mTBI into clinical workflows could refine patient stratification, offering a more nuanced understanding of tumor dynamics than binary ctDNA status alone. Moreover, ctDNA allows for the real-time monitoring of clonal evolution, providing insights into how tumors adapt under therapeutic pressure. Serial ctDNA profiling can detect emerging resistance mutations, such as alterations in the FGFR3, PI3K/AKT/mTOR, or other relevant pathways [49]. This dynamic tracking supports the early identification of resistance, enabling timely therapeutic adjustments, including switching or combining treatment modalities. By capturing tumor evolution at the molecular level, ctDNA contributes to the development of adaptive, personalized treatment strategies that respond to the shifting genetic landscape of the disease.

Despite these challenges, the future of ctDNA in MIBC is promising. Ongoing clinical trials, including IMvigor011, MODERN, and VOLGA, are prospectively evaluating ctDNA-guided treatment strategies and are expected to provide the evidence necessary to support its integration into clinical practice (Table 2). Technological advancements continue to enhance ctDNA detection, with improvements in sequencing sensitivity, machine learning-based data interpretation, and the integration of ctDNA with other biomarkers such as radiomics and proteomics.

An exciting area of development is the emergence of point-of-care ctDNA platforms, which could facilitate rapid, on-site molecular assessments and real-time treatment decisions. These innovations, combined with growing clinician familiarity, are expected to accelerate the adoption of ctDNA as a cornerstone of precision oncology in bladder cancer.

In summary, while ctDNA offers transformative potential for personalizing prognosis and treatment in MIBC, addressing the technical, biological, and logistical challenges is essential. Future research should focus on standardization, validation through prospective trials, and seamless integration into clinical workflows to fully realize the benefits of ctDNA-guided care.

## 6. Conclusions

Circulating tumor DNA has emerged as a transformative biomarker in the management of muscle-invasive bladder cancer, offering unparalleled opportunities for non-invasive, real-time insights into tumor biology. From the preoperative risk assessment and monitoring of minimal residual disease to guiding adjuvant and systemic therapies, ctDNA has the potential to fundamentally reshape clinical practice in MIBC. Current evidence supports its role in improving prognostic accuracy, tailoring therapeutic strategies, and detecting recurrence earlier than conventional methods.

However, several challenges—including standardization, cost, and biological variability—must be addressed before ctDNA can be widely adopted in routine care. The optimal use of ctDNA, including its timing, frequency, and clinical thresholds, remains an area of active investigation. Ongoing trials and technological advances, including the integration of artificial intelligence, are expected to further validate its clinical utility and expand its applications [50]. As precision oncology continues to evolve, ctDNA is poised to become an essential component of personalized care, ultimately improving outcomes and quality of life for patients with bladder cancer.

## Figures and Tables

**Table 1:** Comparison of tumor-informed and tumor-agnostic ctDNA testing methodologies.

Feature	Tumor-Informed Testing	Tumor-Agnostic Testing
Basis of Testing	Patient-specific mutations identified from tumor tissue	Standardized gene panels covering common mutations
Sensitivity	High (especially for MRD detection)	Moderate to low (may miss low ctDNA levels)
Specificity	High (targets known tumor mutations)	Lower (risk of false positives from clonal hematopoiesis)
Turnaround Time	Longer (requires tumor sequencing and assay design)	Faster (no need for tumor tissue sequencing)
Mutation Coverage	Limited to known mutations in the original tumor	Broader, may detect de novo mutations
Clinical Utility	Best for monitoring MRD and treatment response	Useful when tumor tissue is unavailable or rapid testing needed
Cost	Higher (custom assay development)	Lower to moderate
Limitations	Requires tumor sample and more time	Less sensitive; may miss low-frequency variants

**Table 2:** Key clinical studies investigating ctDNA in muscle-invasive bladder cancer (MIBC).

Study/Trial	Design and Setting	Population	Key Findings
Christensen et al.	Prospective observational	68 patients with localized MIBC receiving neoadjuvant cisplatin-based chemotherapy	ctDNA clearance during NAC was associated with a 3-year RFS of 88% versus 46% in patients with persistent ctDNA. ctDNA dynamics predicted pathological response and long-term outcome.
ABACUS (NCT02662309)	Phase II, single-arm trial	95 cisplatin-ineligible MIBC patients received neoadjuvant atezolizumab	ctDNA negativity or clearance post-treatment was associated with pathological complete response and no relapse. Persistent ctDNA predicted increased recurrence risk.
NABUCCO (NCT03387761)	Phase I/II	24 patients with high-risk, operable MIBC treated with neoadjuvant ipilimumab + nivolumab	Patients ctDNA-negative at surgery had 88% PFS at 12 months. Those with detectable ctDNA had significantly poorer outcomes.
IMvigor010 (NCT02450331)	Phase III RCT	809 high-risk post-cystectomy MIBC patients randomized to adjuvant atezolizumab vs observation	Although the trial was negative overall, ctDNA-positive patients derived DFS benefit from atezolizumab (HR 0.58), while ctDNA-negative patients did not.
IMvigor011 (NCT04660344)	Phase III RCT (ongoing)	Post-cystectomy patients with ctDNA+ randomized to atezolizumab or placebo	Aims to validate ctDNA-guided adjuvant immunotherapy. DFS is the primary endpoint in ctDNA + group.
TOMBOLA (NCT04138628)	Phase II (ongoing)	ctDNA-guided surveillance post-cystectomy with atezolizumab initiated upon ctDNA detection	Early data: relapse rate of ~3% among ctDNA-negative patients. Supports risk-adapted surveillance.
MODERN (NCT05987241)	Phase II/III RCT (ongoing)	Postoperative MIBC patients stratified by ctDNA status	CtDNA + patients receive nivolumab ± relatlimab. Study evaluates efficacy of immunotherapy escalation based on molecular risk.
VOLGA (NCT04960709)	Phase III RCT (ongoing)	Cisplatin-eligible MIBC patients receiving neoadjuvant chemo-immunotherapy	ctDNA MRD monitoring integrated as an endpoint. Preliminary findings suggest ctDNA clearance predicts pCR and improved survival.

## Data Availability

Data sharing is not applicable.
